# Early Childhood Caries

**DOI:** 10.1155/2011/725320

**Published:** 2011-10-10

**Authors:** Yumiko Kawashita, Masayasu Kitamura, Toshiyuki Saito

**Affiliations:** Department of Oral Health, Nagasaki University Graduate School of Biomedical Sciences, 1-7-1 Sakamoto, Nagasaki 852-8588, Japan

## Abstract

Dental caries is one of the most common childhood diseases, and people continue to be susceptible to it throughout their lives. Although dental caries can be arrested and potentially even reversed in its early stages, it is often not self-limiting and progresses without proper care until the tooth is destroyed. Early childhood caries (ECC) is often complicated by inappropriate feeding practices and heavy infection with mutans streptococci. Such children should be targeted with a professional preventive program that includes oral hygiene instructions for mothers or caregivers, along with fluoride and diet counseling. However, these strategies alone are not sufficient to prevent dental caries in high-risk children; prevention of ECC also requires addressing the socioeconomic factors that face many families in which ECC is endemic. The aim of this paper is to systematically review information about ECC and to describe why many children are suffering from dental caries.

## 1. Introduction

The term “dental caries” is used to describe the results, signs, and symptoms of a localized chemical dissolution of the tooth surface caused by metabolic events taking place in the biofilms (dental plaque) that cover the affected area [1]. Children in the age range of 12–30 months have a special caries pattern that differs from that in older children. Caries affects the maxillary primary incisors and first primary molars in a way that reflects the pattern of eruption. The longer the tooth has been present and exposed to the caries challenge, the more it is affected. The upper incisors are most vulnerable, while the mandibular incisors are protected by the tongue and by saliva from submandibular and sublingual glands [1]. This pattern of dental caries has been labeled variously as “bottle caries,” “nursing caries,” “baby bottle tooth decay,” or “night bottle mouth.” These terms suggest that the prime cause of dental caries in early childhood is inappropriate bottle feeding. Current evidence suggests that use of a sugar-containing liquid in a bottle at night may be an important etiological factor, although it is not necessarily the only etiological factor. Therefore, it is recommended that the term “early childhood caries (ECC)” be used when describing any form of caries in infants and preschool children [2, 3].

 ECC begins with white-spot lesions in the upper primary incisors along the margin of the gingiva. If the disease continues, caries can progress, leading to complete destruction of the crown [4, 5]. Children experiencing caries as infants or toddlers have a much greater probability of subsequent caries in both the primary [6] and the permanent dentitions [7]. Not only does ECC affect teeth, but the consequences of this disease may also lead to more widespread health issues. Infants with ECC grow at a slower pace than caries-free infants. Some young children with ECC may be severely underweight because of associated pain and their disinclination to eat [8]. ECC may also be associated with iron deficiency [9].

**Figure 1:**
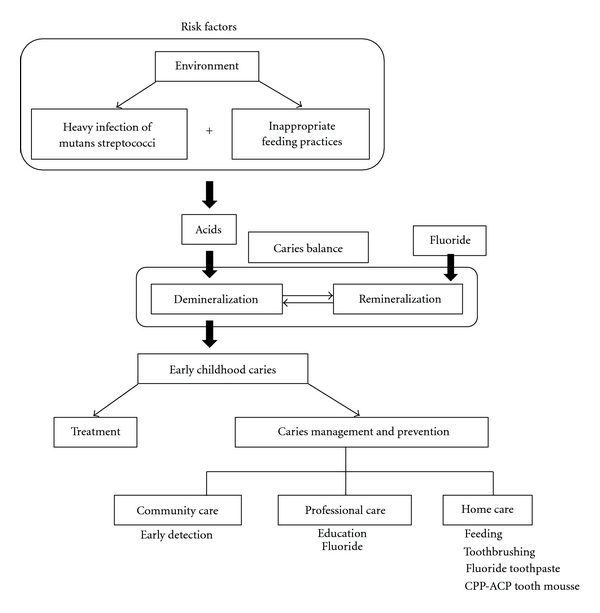
Brief overview of early childhood caries.

 Dental caries is a preventable disease, and it can be stopped and even potentially reversed during its early stages. People remain susceptible to the disease throughout their lives. The objective of this paper is to demonstrate why many children are suffering from dental caries by reviewing published reports on prevalence, process, risk factors, treatment, prevention, and future approaches to prevent ECC (Figure 1).

## 2. Prevalence

ECC is a public health problem that continues to affect infants and preschool children worldwide. A comprehensive review of the epidemiology of ECC showed that its prevalence varies from population to population; however, disadvantaged children, regardless of race, ethnicity, or culture, are most vulnerable. In the United States, the Centers for Disease Control and Prevention (CDC) reported that the prevalence of dental caries among the nation's youngest children, aged 2–5 years, was 24.2% in the National Health and Nutrition Examination Survey (NHANES) III between 1988 and 1994 and 27.9% in NHANES 1999–2004 [10, 11]. Among children aged 2–11 years during 1999–2004, Mexican-American children had higher caries levels (55.4%) than black (43.4%) or non-Hispanic white children (38.6%). Children from families with incomes ≧200% of the federal poverty level (FPL) had a lower caries experience (32.3%) compared to those in lower income groups (48.8% for those with family incomes of 100–199% of the FPL and 54.3% for those with family incomes <100% of the FPL) [10]. 

 In developing countries, the prevalence of ECC differs according to the group examined, and a prevalence of up to 85% has been reported for disadvantaged groups [12, 13]. In the Western world, the prevalence at 3 years of age was 19.9%, and strong associations were found with socioeconomic status and ethnicity [14]. In a Japanese national survey in 2007, the experience of ECC was 2.8% among 18-month-old children and 25.9% among 3-year-old children [15].

## 3. Process

The presence of a fermentable carbohydrate (e.g., sucrose, glucose, fructose, cooked starch) and biofilms on the teeth support the metabolism of acidogenic microorganisms, resulting in acidic substances, the hydrogen ions of which dissolve the carbonated hydroxyapatite crystal lattice of enamel, cementum, and dentin. Continued demineralization results in cavitation of the tooth enamel surface [16]. It is more difficult to remove biofilms from rough, cavitated surfaces, thus potentiating rapid bacterial replication and subsequent growth of bacterial colonies. In the primary dentition, when demineralization passes from the outer enamel tooth layer to the more highly organic dentin layer, caries progression is rapid, and restorative dentistry is often required.

 The body's natural repair mechanism for dental caries, or demineralization, is called remineralization, a process whereby minerals from saliva diffuse back into the porous subsurface region of the demineralized lesion. The cycle of demineralization and remineralization continues throughout the day. When fluoride is present in saliva, it is strongly adsorbed to the demineralized surface of the tooth and protects its crystal surface against acid dissolution. Whether a lesion will progress, remain the same, or becomes reversed is determined by the balance between protective factors and pathological factors, which is called the “caries balance” [16].

## 4. Risk Factors

### 4.1. Microbiological Risk Factors

ECC is an infectious disease, and mutans streptococci (MS), including the species *Streptococcus mutans *and *Streptococcus sobrinus*, are the most common causative agents. Lactobacilli also participate in the development of caries lesions and play an important role in lesion progression, but not its initiation [17]. Diet also plays an important role in the acquisition and clinical expression of this infection. Early acquisition of MS is a key event in the natural history of the disease [18].

 Vertical transmission of MS from caregiver to child has been reported [19]. The major reservoir of MS is the mother, from whom the child acquires it during a window period of around 2 years of age. At this time, the child is probably most susceptible to acquiring MS [19]. Successful infant colonization of maternally transmitted MS may be related to several factors, which include the magnitude of the inoculum, the frequency of small-dose inoculations, and the minimum infective dose. Mothers with dense salivary reservoirs of MS are at high risk of infecting their infants very early in life [20]. Thus, poor maternal oral hygiene and higher daily frequencies of snacking and sugar exposure increase the likelihood of transmission of the infection from mother to child [21]. In addition to maternal transmission of MS, father-to-child transmission has been studied [22]. Horizontal transmission was also examined; transmission of microbes may occur between members of a group (e.g., siblings, toddlers at a nursery) [20].

 According to a recent study, neonatal factors may also increase the risk for early acquisition of *S. mutans* via vertical transmission. Infants delivered by cesarean section acquire *S. mutans* earlier than vaginally delivered infants. The investigators hypothesized that vaginal delivery may expose newborns to early protection against *S. mutans* colonization. That is, by being exposed to numerous bacteria earlier and with great intensity, the pattern of microbial acquisition is affected. Cesarean infants are delivered in a typically more aseptic manner, resulting in an atypical microbial environment that may increase susceptibility to subsequent early *S. mutans* colonization [23].

 The time span between MS colonization and caries lesion development is approximately 13–16 months. In more high-risk children (preterm and/or low-birth-weight infants, with hypomineralized teeth), the duration is likely to be much shorter. Considerable presumptive evidence exists that malnutrition/undernutrition during the prenatal and perinatal periods causes hypoplasia. A consistent association has been reported between enamel hypoplasia and ECC [21, 24].

### 4.2. Dietary Risk Factors

In addition to heavy infection with MS, children with ECC typically experience frequent and prolonged consumption of sugared beverages [25–27]. Sugared beverages are readily metabolized by MS and lactobacilli to organic acids that can demineralize enamel and dentin. The use of nursing bottles enhances exposure to lactose.

 Cow milk in a nursing bottle is often assumed, incorrectly, to be a primary causative agent in the induction of ECC [28]. Available experimental evidence *in vivo* and *in vitro* clearly shows that cow milk has negligible cariogenicity. Indeed, cow milk is essentially noncariogenic because of its mineral content and low level of lactose [25, 26, 28–30]. Saliva production decreases during sleep, and the protracted presence of a teat or nipple can result in promoting the cariogenic potential of the fluid part of an infant's diet. Thus, water should be the only drink given to a child during the night [1].

 The cariogenicity of human milk is the subject of some controversy. A systematic review of epidemiological evidence suggests that breast feeding for longer than 1 year and at night may be associated with an increased prevalence of dental decay [31]. Also, a study demonstrated that human milk promoted the development of smooth-surface caries and was significantly more cariogenic than cow milk. However, no significant difference in the caries scores of the sulcal surfaces of the cow milk and human milk groups was detected [26]. Moreover, an epidemiological study demonstrated that breast feeding and its duration were independently associated with an increased risk for ECC and a greater number of decayed or filled tooth surfaces among children aged 2–5 years in the United States [32]. However, it should also be noted that these children were living in poverty.

### 4.3. Environmental Risk Factors

A systematic review concluded that children were most likely to develop caries if MS was acquired at an early age, although this may be partly compensated for by other factors, such as good oral hygiene and a noncariogenic diet [3]. Development of oral hygiene habits may be sensitive to the economic environment in which children live. Such environmental factors include caregivers' social status [33–35], poverty, ethnicity, deprivation, number of years of education, and dental insurance coverage. Despite the widespread decline in caries prevalence and severity in permanent teeth in high-income countries over recent decades, disparities remain, and many children still develop dental caries [36, 37]. This relatively new area of research has been called “life-course epidemiology” [38]. The life-course framework for investigating the etiology and the natural history of chronic diseases proposes that advantages and disadvantages are accumulated throughout life, generating differentials in health along the life course and leading to large effects in later life.

 Children with a history of dental caries, whose primary caregiver or siblings have severe dental caries, are regarded as being at increased risk for the disease [37, 39]. Moreover, children's experience of socioeconomic disadvantage affects adult dental health [40]. However, a cross-sectional study in Japan reported that dental caries in 3-year-old children was more strongly associated with child-rearing behaviors than mother-related factors, such as health insurance, health behaviors, and dental health status [41].

## 5. Treatment

Determining the causes of dental caries in children, providing education on oral health matters to their parents or caregivers, and controlling demineralization are especially important because children's cooperative capacity is low. Interventions aimed at improving the intraoral environment can reduce the risk of dental caries and can arrest dental caries.

 Treatment sometimes consists of restoration or the surgical removal of carious teeth. However, this approach does little to bring the disease under control because the recurrence of caries around restored teeth and the occurrence of further decay are common [18, 42]. Relapse rates of approximately 40% within the first year after dental surgery have been reported. Thus, dental caries management in many countries has shifted toward a largely preventative and preservative approach rather than surgical treatment. Prevention and preservation of tooth tissue are desirable as the normal treatment for dental caries because we know that dental caries progresses slowly in most people, prevention is effective, and excessive and premature surgical treatment can cause harm [43–45]. When restorative intervention is needed, modern microrestorative techniques that use new adhesive materials can also preserve tooth structure [46].

## 6. Prevention

### 6.1. Target Cariogenic Feeding and Primary Acquisition of MS

Prevention of cariogenic feeding behavior is one approach for preventing ECC. Sugared beverage consumption with nursing bottles or “sippy cups” enhances the frequency of enamel demineralization. This type of feeding behavior during sleep intensifies the risk of dental caries because oral clearance and salivary flow rates decrease during sleep. Thus, sugared beverage consumption with nursing bottles should be reduced or stopped.

 Also, the knowledge that the most important risk factor related to dental caries in babies is acquisition of MS should help in determining an optimal preventive approach and interceptive treatment. A promising approach toward primary prevention of ECC is the development of strategies that target the infectious component of this disease, such as preventing or delaying primary acquisition of MS at an early age through suppressing maternal reservoirs of the organism.

 For this reason, it is better if prevention of ECC begins in the prenatal and perinatal periods (including pregnancy and the first month after birth) and addresses the health of both the mother and the infant. The mother's or caregiver's teeth should be examined. Infants whose mothers have high levels of MS due to untreated dental decay are at greater risk of acquiring the organisms. Dental management of the mother can delay infant inoculation [47].

### 6.2. Topical Antimicrobial Therapy

Topical antimicrobial therapies have been recently described. Topical application of a 10% povidone-iodine solution to the dentition of infants every 2 months in a double-blind, placebo-controlled clinical trial for 1 year increased the number of caries-free infants [48]. These infants were at high risk for ECC as they were all colonized by MS and had decay-promoting feeding behaviors. This study suggested that povidone-iodine had suppressive effects on the oral colonization of MS and prevented dental caries. However, povidone-iodine has strong bactericidal/virucidal effects and demolishes normal flora in the pharynx and the oral cavity, which interfere with pathogenic viral invasion [49]. Therefore, povidone-iodine should not be routinely used.

 In another study, 6 monthly applications of a 40% chlorhexidine varnish were effective in a 37.3% reduction in caries increment without side effects [50], and this reduction was also close to that found in a meta-analysis regarding the effectiveness of fluoride varnish on caries prevention in primary teeth, 33% (95% CI = 19–58%) [51]. Topical 0.12% chlorhexidine gluconate could significantly reduce MS levels, but chlorhexidine therapy was much less effective at reducing the levels of lactobacilli in the human mouth. Current chlorhexidine products require patient compliance with a rinse that tastes bad and has the potential to stain, and it must be applied numerous times to be effective [52]. Moreover, a systematic review reported that the evidence for a caries-preventive effect of chlorhexidine varnish in children and adolescents was inconclusive [53].

### 6.3. Fluoride

To prevent ECC by home-care approaches, brushing by caregivers using a small quantity of fluoride-containing toothpaste is essential and should start as soon as teeth erupt. Pine et al. [54] showed the benefit of twice daily brushing in newly erupted first molar teeth compared to brushing once daily or less. This study also showed the importance of parental beliefs. If parents feel strongly that there is time to check the condition of their child's teeth, the odds that their child will actually brush twice daily are about three times greater. Thus, it is important to support parents and convince them that their efforts make sense for their child's dental health and that they really can contribute.

 Moreover, community and professional care approaches have been used to prevent ECC [55]. Early screening for signs of dental caries development, starting from about 7-8 months of age, could identify infants who are at risk of developing ECC, assist in providing information for parents about how to promote oral health, and prevent the development of tooth decay. High-risk infants include those with early signs of ECC, poor oral hygiene (of both infant and mother), limited exposure to fluoride, and frequent exposure to sweet beverages. These infants should be targeted with a professional preventive program that includes oral hygiene instructions for the mother and child, fluoride use, and diet counseling. These professional approaches are important but not sufficient to prevent dental caries in high-risk children. Addressing the social and economic factors that many families face where ECC is endemic is also necessary [55].

### 6.4. Casein Phosphopeptide-Amorphous Calcium Phosphate (CPP-ACP)

CPP-ACP nanocomplexes are casein-derived peptides in which ACP is stabilized by CPP. These nanocomplexes act as a calcium and phosphate reservoir when incorporated into the dental plaque and on the tooth surface [56]. CPP-ACP has been shown to reduce demineralization and promote remineralization of carious lesions both *in vitro* [57] and *in situ* [58] and to reduce erosive tooth wear *in vitro* [59]. CPP-ACP cream, which is effective in remineralizing early enamel lesions of primary teeth, was a little more effective than 500 ppm NaF [60]. Moreover, CPP-stabilized amorphous calcium fluoride phosphate had a greater remineralizing effect on carious lesions compared to fluoride or CPP-ACP individually. Since additive effects were obtained when CPP-ACP was used in conjunction with fluoride, CPP-ACP is better used as a self-applied topical coating after the teeth have been brushed with a fluoridated toothpaste by children who have a high risk of dental caries [61].

### 6.5. Pediatricians' Role

Prevention and control of dental caries can be promoted by clinicians other than dentists if such clinicians are appropriately trained [37, 46, 62]. Pediatricians can provide recommendations for the prevention of ECC to mothers and caregivers. Children can be examined by their primary care provider or pediatrician for signs of early carious demineralization, as indicated by white areas around the gingival margin or brown-stained pits and fissures. The detection of dental caries and referral to an appropriate dental care professional for treatment should be thought of as a secondary prevention measure.

### 6.6. Dental Fluorosis

Two studies have been published supporting the effectiveness of fluoride varnish to prevent dental caries in the primary dentition [63, 64]. However, fluoride varnish can also introduce the risk of the development of enamel fluorosis in the permanent teeth [65–67]. Evidence of a major benefit from fluoride consumption during infancy is lacking, and thus, it seems reasonable to limit the intake of fluoride to less than 70 *μ*g/kg BW per day, considering the possible risk of enamel fluorosis [68]. To avoid greater intake, water with relatively low fluoride content (e.g., 0–0.3 mg/L) is recommended to be used as a diluent for infant formula, and no fluoride supplement should be given to infants.

 For children 1–7 years of age, the repeated addition of small amounts of fluoride to oral fluids is important [68]. Consumption of fluoridated water is highly recommended, and the regular use of fluoridated dentifrices is also an effective means of decreasing the prevalence of dental caries. However, with the knowledge that small children swallow much of the applied dentifrice, education regarding appropriate tooth brushing in small children is needed for mothers or caregivers. The recommended limit in the amount of dentifrice should be no more than 0.25 g per brushing [68].

 Fluoride supplements have been recommended for preventing caries. A systematic review [69] found that the evidence supporting the effectiveness of supplements in caries prevention in primary teeth was weak. In permanent teeth, the daily use of supplements prevented dental caries. The use of supplements during the first 6 years of life, and especially during the first 3 years, was associated with a significant increase in fluorosis.

## 7. Future Approaches to Prevent ECC

Considering the integrated roles of dental, medical, and other health care providers, assessing the effects of public health interventions, and introducing oral health promotion as part of general health promotion are all necessary [46]. The mouth can be both a nidus of infection and the location of the first sign of systemic disease, and pediatricians have frequent access to young children and have opportunities to address issues relating to oral health. Thus, primary care clinicians should be familiar with effective interventions for the youngest children before they require dental services. A study demonstrated that oral health training during residency can increase pediatrician confidence in participating in important oral health-promoting tasks, including anticipatory guidance, oral screenings, and oral health-risk assessments [62].

 Additionally, dentists need to establish the best ways to provide preventive and clinically effective care. Scientific advances must blur the demarcation between dental and medical practices; dental caries is a health problem that can be managed by a team of health care providers including dentists and physicians [70]. Physicians must concentrate on using existing methods to detect signs of early and advanced caries and provide advice on how to prevent and control caries in their patients.
